# A rare case of atrioventricular block

**DOI:** 10.1007/s12471-021-01588-1

**Published:** 2021-06-08

**Authors:** R. Eerdekens, L. M. Rademakers

**Affiliations:** Department of Cardiology, Catharina Hospital Eindhoven, Eindhoven, The Netherlands

**Fig. 1:**
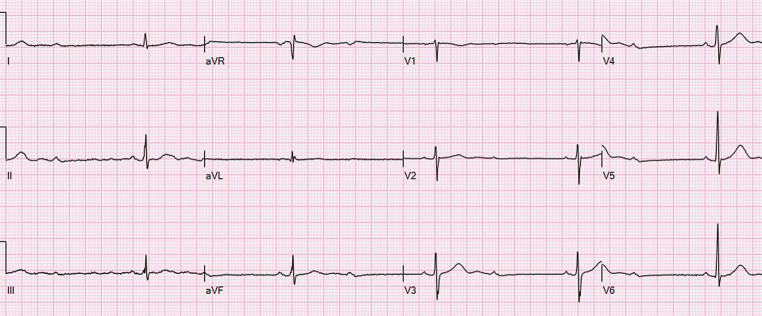
Electrocardiogram showing sinus rhythm with 2:1 atrioventricular block

**Fig. 2:**
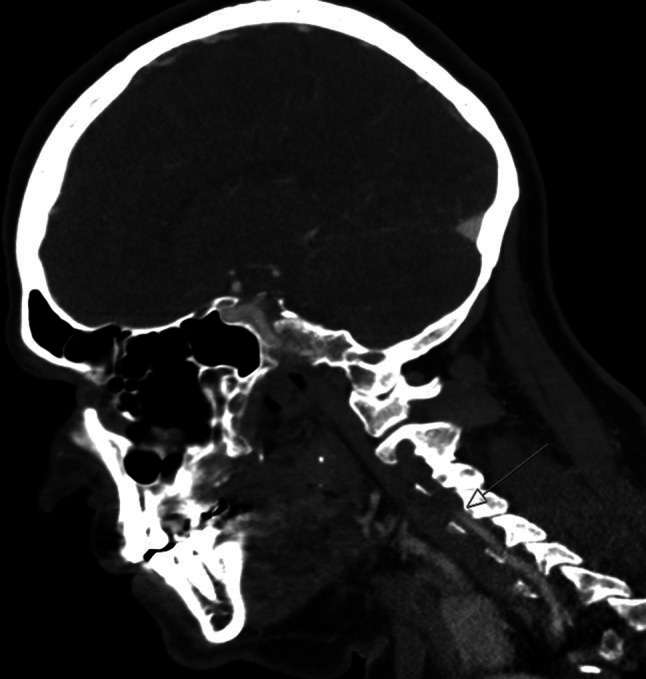
Computed tomography, sagittal view. *Arrow* shows occluded artery

**Fig. 3:**
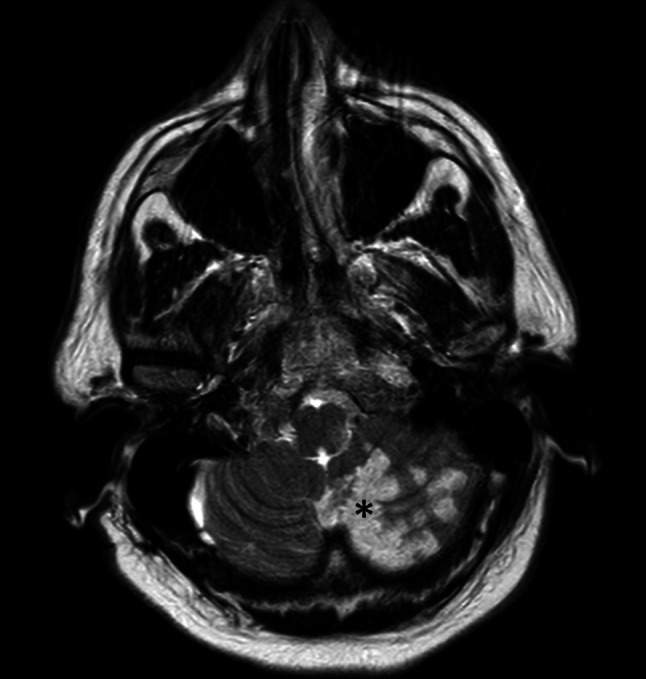
T2-weighted magnetic resonance imaging showing infarcted area (*asterisk*)

A previously healthy 57-year-old male presented at the emergency department (ED) with sudden onset of dizziness followed by nausea. Episodes lasted for approximately 10 min and resolved spontaneously. At the ED, while the patient was asymptomatic, clinical (including neurological) examination, laboratory test, electrocardiogram and echocardiography were normal. He suddenly experienced dizziness while the electrocardiogram showed sinus rhythm with 2:1 atrioventricular (AV) block (Fig. 1). The patient was diagnosed with symptomatic Mobitz type II AV block and was admitted for pacemaker implantation. On the ward he again experienced dizziness but now accompanied by neurological symptoms, i.e. ataxia, nystagmus, miosis and ptosis. Urgent cerebral computed tomography angiography showed an acute occlusion of the left-sided posterior inferior cerebellar artery (PICA) (Fig. 2). He was treated with thrombolysis, recovered well and AV block was not recorded anymore. Thereafter, magnetic resonance imaging (Fig. 3) revealed a large infarcted area related to the PICA.

Conduction disturbances, caused by autonomic dysregulation, are uncommon during acute ischaemic stroke, but can be seen in lateral medullary syndrome (Wallenberg or PICA syndrome) [1, 2].
